# Vaginal and laparoscopic sub-urethral sling explantation

**DOI:** 10.1007/s00192-023-05495-4

**Published:** 2023-03-11

**Authors:** Virginie Collin-Bund, Victor Gabriele, Chris Minella, Lise Lecointre, Thomas Boisramé, Emilie Faller, Aline Host, Chérif Akladios, Christian Saussine, Olivier Garbin

**Affiliations:** 1 Department of Gynecologic Surgery, Hôpitaux Universitaires de Strasbourg, 67091 Strasbourg, France; 2 Laboratoire d’ImmunoRhumatologie Moléculaire, Institut National de la Santé et de la Recherche Médicale (INSERM) UMR_S 1109, Institut thématique interdisciplinaire (ITI) de Médecine de Précision de Strasbourg, Transplantex NG, Faculté de Médecine, Fédération Hospitalo-Universitaire OMICARE, Fédération de Médecine Translationnelle de Strasbourg (FMTS), Université de Strasbourg, Strasbourg, France; 3 Department of Urology Surgery, Hôpitaux Universitaires de Strasbourg, 67091 Strasbourg, France

**Keywords:** Suburethral sling, Vaginal surgery, Laparoscopic surgery, Retzius space

## Abstract

**Introduction and hypothesis:**

The objective was to describe the different laparoscopic and vaginal steps of sub-urethral infected mesh explantation as well as an unexpected and unusual complication: a sub-mucosal calcification on the sub-urethral segment of the sling that was not infiltrating the urethra.

**Methods:**

This was carried out at our University Teaching Hospital of Strasbourg.

**Results:**

We show the complete removal of an infected retropubic sling in a patient who had already undergone three previous surgeries without resolution of symptoms. This is a difficult case requiring a laparoscopic approach of the space of Retzius, which has been less familiar to surgeons since the advent of the midurethral sling. We show how to approach this space in an inflammatory environment by specifying its anatomical limits. Moreover, a great deal can be learned from the occurrence of an infectious complication after the surgery and the presence of a large calcification on the prosthesis. In this context, we advise a systematic antibiotic treatment to avoid this kind of complication.

**Conclusions:**

Knowing the guidelines and the different surgical steps will help urogynecological surgeons to perform similar procedures in patients requiring removal of retropubic slings for complications such as infection and pain, where conservative management has not been successful. These cases must be discussed in a multidisciplinary meeting, as recommended by the French National Authority for Health, and managed in an expert establishment.

**Electronic supplementary material:**

The online version of this article (10.1007/s00192-023-05495-4) contains supplementary material. This video is also available to watch on http://link.springer.com/. Please search for this article by the article title or DOI number, and on the article page click on ‘Supplementary Material’

The aim of this video is to describe the different laparoscopic and vaginal steps of sub-urethral infected mesh explantation as well as an unexpected and unusual complication: sub-mucosal calcification on the sub-urethral segment of the sling that was not infiltrating the urethra.

Our case is that of a 57-year-old patient with a medical history of non-insulin-dependent type 2 diabetes, hypertension, and obesity. Surgically, she underwent gastric bypass surgery in 2014 followed by an abdominoplasty. With regard to her gynecological and obstetrical history, an endometrial thermocoagulation was performed in 2005 followed by a vaginal hysterectomy in 2007. She had undergone four vaginal deliveries. In 2000, a retropubic sling was been applied by a vaginal approach as part of treatment for urinary incontinence. In 2011, the sling had to be sectioned because of chronic urinary retention, followed in 2012 by seven surgeries to treat a vulvar foramen granuloma as well as a thread sepsis. Thereafter, the patient presented with several episodes of urinary tract infection, chronic bladder retention, and dirty vaginal discharge. On endovaginal ultrasound, the sling was abnormally visible and visualized a left para-urethral tubular formation with a blocked end in the left parasymphysis. MRI examination showed the sling without bladder invasion but with a significant post-micturition residue (200 cc). Hyperintensity under the bladder indicated by the green arrow seemed to correspond to a suspected abscess. An urethroscopy was performed, which was normal. Considering the symptomatology and radiological data, we decided to explant the sling.

A mixed surgical (vaginal and laparoscopic) approach was then planned. The entire surgical procedure was supervised by a per-operative antibiotic prophylaxis according to current guidelines. The operation was started vaginally. A calcification of about 2 cm long was discovered, which was very adherent to the band. The calcification was gripped with a Babcock forceps and the strip sectioned, taking the whole calcification with it owing to intense adhesion. A blue bladder test was performed, which did not reveal any lesion or bladder infiltration. The retropubic part of the sling, in the space of Retzius, was not accessible via a vaginal approach. A laparoscopic approach was then performed. The first step consisted in dissecting the umbilical and pre-vesical fascia. Dissection was progressive until the pubic symphysis was reached. Dissection was extended to the space of Retzius, which was difficult to access owing to an inflammatory environment. This was a difficult case requiring a laparoscopic approach of the space of Retzius, which has been less familiar to surgeons since the advent of midurethral slings and the decrease in the frequency of the Burch procedure [1].

The anatomical limits of the space of Retzius are defined as follows: the upper limit by the umbilical and prevesical fascia opened initially, the symphysis pubis and Cooper's ligament, the medial part by the obturator internus muscle and the tendinous arch of the levator ani muscle, and the lower part bounded by the bladder.

On the 6th day post-operatively, the patient was referred to the emergency department with fever associated with pelvic pain. Clinically, there was minimal swelling of the right lateral vaginal wall. Biology showed a major inflammatory syndrome and ultrasound examination found a 60- to 72-mm pre-vesical abscess without effusion in the Douglas space. An emergency CT scan was performed confirming a 7- to 9-cm abscessed collection of the right space of Retzius, infiltrating the right vaginal wall. In this context, a surgical intervention with a drainage of the abscess was performed. A drain was put in vaginally and removed 4 days later. At the same time, intravenous antibiotic therapy with ceftriaxone and metronidazole was introduced. In retrospect, given the calcification, the periprosthetic capsule, and the intraoperative inflammation, it is thought that antibiotic prophylaxis within 5 days of the operation could have prevented this complication. Post-operative follow-up 6 weeks later was uneventful. The patient had no complaints other than grade 1 stress urinary incontinence and described good bladder emptying without associated chronic retention.

Knowing the difficulty of this case and the expected surgery, it is essential to have a multidisciplinary team and to be able to discuss these cases in a multidisciplinary consultation meeting. According to the latest report of the French High Authority, dated October 2020 [2], a multidisciplinary consultation must be set up before any implantation, and not only in so-called complex forms of urinary incontinence [3]. It is essential that all the solutions for the management of female stress urinary incontinence are systematically considering patients’ clinical condition. In addition, the use of this type of medical device should be reserved for centers capable of ensuring all stages of patient management, from the initial evaluation to implantation and post-implantation follow-up. In fact, management of serious post-implantation complications should be, if necessary, the subject of a multidisciplinary consultation. The final decision should be shared and discussed with the patient after consideration of all the information and sufficient time to reflect. If an explantation is necessary, the procedure should be performed in a center with a multidisciplinary surgical facility and trained surgeons.

## Supplementary information

ESM 1 (MP4 112334 kb)
